# Role of YY1 in the Regulation of Anti-Apoptotic Gene Products in Drug-Resistant Cancer Cells

**DOI:** 10.3390/cancers15174267

**Published:** 2023-08-25

**Authors:** Megan Jung, Indy Bui, Benjamin Bonavida

**Affiliations:** Department of Microbiology, Immunology & Molecular Genetics, David Geffen School of Medicine, Jonsson Comprehensive Cancer Center, University of California, Los Angeles, CA 90095, USA

**Keywords:** cancer resistance, YY1, Bcl-2, Bcl-xL, Mcl-1, survivin, YY1 inhibitors, molecular regulation

## Abstract

**Simple Summary:**

New treatments that utilize targeted therapeutic agents or invigorations of the host immune system to destroy cancer cells are not effective against all cancers or responding patients. The mechanism underlying this failure is the ability of cancer cells to escape destruction and remain resistant to therapy. Understanding the gene products that make cancer cells resistant is beneficial in developing new drugs that inhibit resistant factors. One factor controlling the resistance in cancer cells is the gene regulator or transcription factor (TF) Yin Yang 1 (YY1). High levels of YY1 were detected in resistant cancer cells, while the inhibition restored sensitivities to cytotoxic therapies. This review discusses how YY1 overexpression regulates death protective proteins that control cancer cell resistance. Targeting YY1 in cancer cells for its inhibition or targeting its protective proteins will reverse resistance and allow for cancer cells to respond to therapies, tumor regression and the prolongation of survival.

**Abstract:**

Cancer is a leading cause of death among the various diseases encountered in humans. Cancer is not a single entity and consists of numerous different types and subtypes that require various treatment regimens. In the last decade, several milestones in cancer treatments were accomplished, such as specific targeting agents or revitalizing the dormant anti-tumor immune response. These milestones have resulted in significant positive clinical responses as well as tumor regression and the prolongation of survival in subsets of cancer patients. Hence, in non-responding patients and non-responding relapsed patients, cancers develop intrinsic mechanisms of resistance to cell death via the overexpression of anti-apoptotic gene products. In parallel, the majority of resistant cancers have been reported to overexpress a transcription factor, Yin Yang 1 (YY1), which regulates the chemo-immuno-resistance of cancer cells to therapeutic anticancer cytotoxic agents. The relationship between the overexpression of YY1 and several anti-apoptotic gene products, such as B-cell lymphoma 2 protein (Bcl-2), B-cell lymphoma extra-large (Bcl-xL), myeloid cell leukemia 1 (Mcl-1) and survivin, is investigated in this paper. The findings demonstrate that these anti-apoptotic gene products are regulated, in part, by YY1 at the transcriptional, epigenetic, post-transcriptional and translational levels. While targeting each of the anti-apoptotic gene products individually has been examined and clinically tested for some, this targeting strategy is not effective due to compensation by other overexpressed anti-apoptotic gene products. In contrast, targeting YY1 directly, through small interfering RNAs (siRNAs), gene editing or small molecule inhibitors, can be therapeutically more effective and generalized in YY1-overexpressed resistant cancers.

## 1. Introduction

Yin Yang 1 (YY1) is a transcription factor of the GLI-Kruppel gene family ubiquitously expressed in humans and is known to regulate the activation and repression of transcription [1]. As a DNA-binding transcription factor, YY1′s activator or repressor activity is gene dependent. This includes histone deacetylation, the acetylation of YY1 domains and negative feedback loops [2]. Its activity may also be context-dependent as YY1 interacts with DNA-binding cofactors that recruit promoters to increase transcription [3,4]. It may recruit co-repressors that repress cell differentiation and transforming growth factor-beta (TGF-Β) signaling [5,6]. The zinc-finger region in the DNA-binding domain of YY1 overlaps with transcriptional repression sequences, resulting in the YY1 repressor activity [7]. Additionally, YY1 is involved with controlling other cellular mechanisms, such as cell proliferation, apoptosis and differentiation [8]. YY1 also plays a large role in neurogenesis as well as neurodegenerative diseases of the central nervous system [7]. Due to YY1′s role as a regulator of many genes, it has been shown to have implications with tumor growth as a promoter or suppressor. Most commonly, YY1 displays overexpression in various cancers and is positively correlated with the pathogenesis of cancer [9]. For example, YY1 acts as a prognostic biomarker after its repression of the Raf kinase inhibitory protein (RKIP) in lung cancer [6]. The overexpression of YY1 is linked to tumor cell resistance to cell-mediated immunotherapies [3]. Therefore, YY1 is an attractive drug target for cancer models through preventing DNA binding in order to reduce YY1 expression [9]. Its direct inhibition may block tumor growth in cancer and allow for anticancer drug resistance to be reversed [10].

**Figure 1:**
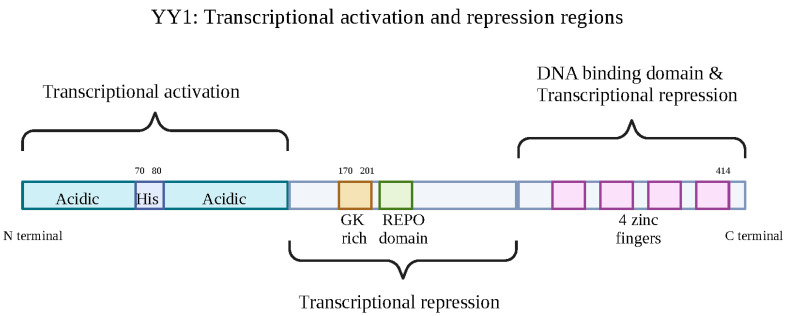
YY1′s activation and repression regions. Transcription factor YY1′s various regions. Made of 414 residues, its N terminal is responsible for transcriptional activation, consisting of two acidic regions and a cluster of histidine proteins. This is followed by a transcriptional repression portion containing a GK rich and REPO domain. Finally, its C-terminal, composed of 4 zinc fingers, is the DNA-binding domain as well as transcriptional repression. Created with BioRender.com (accessed on 1 August 2023).

The transcription factor YY1 consists of 414 amino acids with a molecular weight of ~65,000 Daltons and is encoded on chromosome 14 in humans [11]. Homodimers via the C-terminal domain of YY1′s four C2H2 zinc fingers bind the DNA sequence (5′-CCGCCATNTT-3′) found in enhancers and promoters [7]. Therefore, YY1 acts as an important structural regulator of enhancer-promoter loops [12]. YY1 has influence in both activation and repression as the DNA zinc-binding fingers overlap with transcriptional repression sequences and the transcriptional activator domain located in its N-terminal region [7]. The C-terminal DNA-binding domain with the four zinc fingers each contains two cysteines, whereas the N-terminal region of YY1 consists of a histidine cluster (11 histidines) and two acidic regions [11]. There is also an oncoprotein-binding (OPB) domain made of 26 amino acids that are responsible for YY1′s interaction with the oncogenes murine double minute 2 (MDM2), enhancer of zeste 2 polycomb repressive complex 2 subunit (EZH2), enzyme immunoassay (E1A) and protein kinase B (PKB/AKT) [13]. Therefore, YY1 has the ability to promote oncogenic proliferation in cancer cells. YY1′s involvement in the cell cycle G1/S phase transition is reflected by its subcellular localization. During the entry of the S phase, YY1 exhibits nuclear localization; however, during the second half of the S phase, YY1 localization changes to the cytoplasm [14] (Figure 1).

In terms of transcriptional activity, YY1 can activate transcription through interaction with other partners. Studies have found that the YY1-activating transcription factor 6 (YY1-ATF6) interaction will increase the activity of the transcription of gastrin-releasing peptide (*GRP*) genes [15]. Additionally, YY1 interaction with INOsitol-requiring mutant 80 (INO80) helps to bind target promoter sites that are associated with cell division, DNA replication, genomic stability and DNA repair [16]. YY1 also interacts with the specificity protein 1 (Sp1), causing the upregulation of the Mu Opioid Receptor (*MOR*) gene that is responsible for lymphocyte proliferation suppression. Conversely, YY1 can act as a transcriptional inhibitor through interactions with proteins [17]. For example, YY1 interacts with the protein nuclear factor-kappa B (NF-κB) as a transcriptional repressor of the proapoptotic Bcl-2-interacting mediator of cell death (BIM) through the formation of a YY1–RelA complex [18]. Ultimately, YY1′s activator or repressor characteristic depends on the different interactions with cell type-specific factors.

In this paper, we discuss the regulation of YY1 overexpression in cancer cells, the role of YY1 in the maintenance of cancer resistance to cytotoxic drugs/cells and the role of YY1 in the regulation of anti-apoptotic gene products. The findings demonstrate that YY1 regulates the expression of the anti-apoptotic gene products examined at both the transcriptional and post-transcriptional levels. We present various approaches that target YY1 or anti-apoptotic gene products to reverse resistance as well as future directions in targeting YY1 clinically.

## 2. Regulation of YY1 Expression

### 2.1. Transcriptional Regulation

On a transcriptional level, YY1 acts as an autoregulating transcription factor by its own internal DNA-binding sites. These sites are located within the first intron and are essential for YY1′s promoter transcriptional activity. Therefore, this endogenous gene controls the reduction in and overexpression of the *YY1* gene, leading to variable YY1 protein levels based on homeostatic control [19]. Kim et al. [19] identified three unique self-regulation processes of YY1, namely, (1) evolutionary conservation of the YY1 binding site localized to the first intron of the YY1 locus in vertebrates that allows for its ubiquitous expression in many cellular mechanisms, (2) the protein YY1 is the product of this conserved *cis*-regulatory region and its levels determine the role of repressor or activator during YY1 binding based on homeostatic conditions (high protein levels of YY1 will induce a repressor role during induction and vice versa), and (3) the multiplicity feature of the YY1-binding sites allows for sensor functions in identifying cellular YY1 protein levels through blocking and stimulating RNA polymerase II (Pol II).

**Figure 2:**
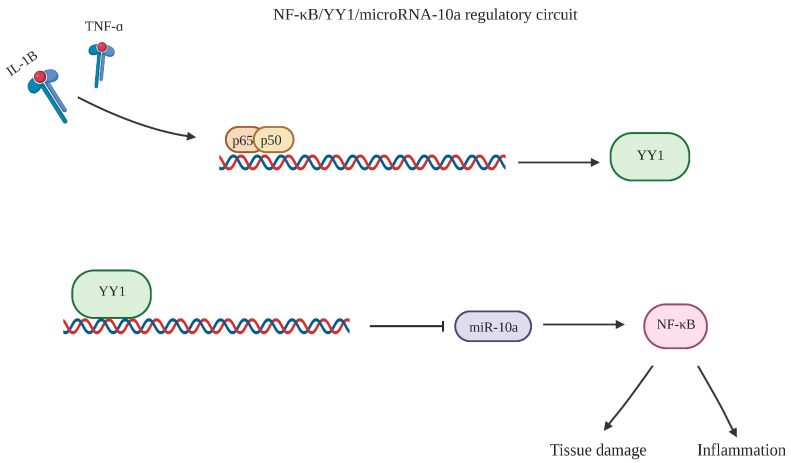
NF-κB/YY1/microRNA-10a regulatory circuit. TNF-α and IL-1Β are activators that cause the p65/p50 heterodimer of NF-κB. This further activates the production of YY1, which goes on to produce downstream effects. Its ability to inhibit miR-10a ultimately increases NF-κB levels in a feedback loop that causes further tissue damage and inflammation in patients with RA. Created with BioRender.com (accessed on 1 August 2023).

The transcriptional regulation of YY1 is also tied to NF-κB as summarized in the study by Wang et al. [20]. The acetylation of RelA(p65) combined with p50 creates the heterodimer of NF-κB [21]. NF-κB regulation then occurs through the direct binding of p65/p50 subunits to the YY1 promoter [20]. In examining the relationship between NF-κB and YY1, Mu et al. examined patients with rheumatoid arthritis (RA) and found that tissue injury was marked by consistent NF-κB activation from tumor necrosis factor-alpha (TNF-α) and interleukin-1 beta (IL-1β). This activation leads to the production of YY1 that downregulates microRNA (miR)-10a, which then activates NF-κB and causes further tissue damage and inflammation [22]. The regulatory circuit NF-κB/YY1/microRNA-10a reveals the activation role of NF-κB in YY1 expression (Figure 2).

RKIP is also involved in the NF-κB regulation pathway but plays a role in the inactivation of YY1. Through several signaling pathways involving multiple interactions and cascades, it was found that RKIP targets the inhibition of YY1 and can be targeted for possible cancer treatments [10]. This is due to the fact that YY1 and RKIP exhibit an inverse relationship by which they are interconnected and, thus, able to alter expression through regulatory loops [23]. Particularly, the NF-κB/Snail/YY1/RKIP loop, which is dysregulated in cancer, reveals that, while the overexpression of NF-κB and Snail causes YY1 activation, the eventual induction of RKIP expression downregulates its precursors: NF-κB, Snail and YY1 [24].

### 2.2. Post-Transcriptional Regulation

**Figure 3:**
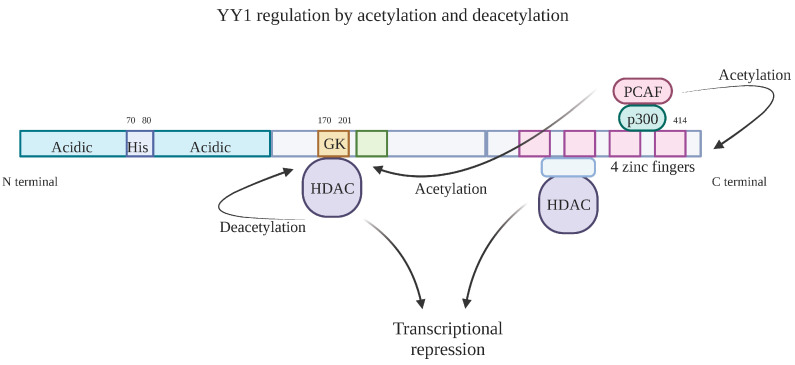
Diagram of the post-translational regulation of YY1 through acetylation and deacetylation. YY1 is subject to deacetylation by the HDAC complex through a negative feedback mechanism in the same region of acetylation from the PCAF and p300 complex. The PCAF/p300 complex located on the DNA-binding domain also acetylates the N-terminal region of YY1, causing an increase in transcriptional repression. YY1′s association with HDAC also results in the formation of a repressor complex. Created with BioRender.com (accessed on 1 August 2023).

The regulation of YY1 can be traced back to its two domains of repression: (1) within residues 170–200 and (2) the overlapping region of the C-terminal zinc finger-binding domain. Whereas its activation domain is located independently at the N-terminus region, post-translational YY1 expression is regulated by acetyltransferase p300/CBP-associated factor (PCAF) acetylations, which occurs by the interaction between acetyl groups and YY1′s many lysine residues, resulting in the augmentation of transcriptional repressor activity [25]. There is also a negative feedback mechanism by which YY1 is subject to deacetylation by histone deacetylase 1 (HDAC1) and HDAC2 in the same central region where acetylation occurs. In the C-terminal zinc finger region, YY1′s HDAC forms a repressor complex that results in the decrease in DNA-binding activity [26]. Additionally, YY1 is regulated through the post-translational modification of methylation. Methyltransferase SET7/9 (KMT7) methylates at YY1′s lysine residues K173 and K411, which ultimately regulate its DNA-binding activity and cell proliferation activity [27]. Regulation can also be traced to the phosphorylation of YY1′s DNA-binding domain. Specifically, it was found that the phosphorylation of YY1 at threonines 348 and 378 leads to the loss of DNA-binding activity during mitosis, which can only be restored upon dephosphorylation [28,29]. Therefore, the phosphorylation of the DNA-binding domain sites can be targeted to modulate YY1 expression in DNA (Figure 3).

### 2.3. Epigenetics and mRNA Regulation

The expression of YY1 in cancer is regulated through various cancer factors and signaling pathways. For example, the E3 ubiquitin ligase Smurf2 mediates YY1 degradation and ubiquitination, which lead to antitumor growth activity [30]. On the other hand, miR-193a-5p downregulation leads to YY1 overexpression in endometrial and lung cancers due to its involvement in migration and metastatic cancer activities [31]. Angiogenesis, which promotes cancer metastasis and the progression of the disease, has been found to be positively correlated with the dysregulation of the Ki-ras2 Kristen rat sarcoma viral oncogene homolog/YY1/zinc finger protein 322/sonic hedgehog signaling molecule (*Kras*/YY1/ZNF322/Shh) transcriptional axis [32]. In patients with pancreatic ductal adenocarcinomas (PDACs), the high prevalence of KRAS activation leads to NF-κB signaling, which activates YY1 and represses miR-489. Therefore, the signaling axis KRAS/NF-κB/YY1/miR-489 leads to PDAC cell metastasis and migration enhancement [33]. Another axis, the stromal cell-derived factor-1 (SDF-1a)/cxc motif chemokine receptor 4 (CXCR4), has been found to increase YY1 in acute myeloid leukemia (AML) cells [34]. YY1 in leukemia has been found to play a role in chemo-resistance through inducing the expression of multidrug resistance 1 gene (*MDR1*) [35]. The miR-7 is also involved in directly targeting YY1 through negative regulation and acts as a tumor suppressor as it stimulates apoptosis and cell-cycle control [36].

### 2.4. Cytoplasm YY1 Stability

During mitosis, YY1′s localization to the cytoplasm excludes it from interacting with DNA from prophase until the beginning of telophase, where it associates with chromosomes in daughter cells [29]. This suggests that YY1 plays a role in the early regulation of active and repressed genes during cell division. YY1 acts as the post-translational modification substrate that inactivates both DNA-binding activity and transcriptional activity [37].

YY1 stability is regulated by the post-translational modification of O-linked N-acetylglucosaminylation (O-GlcNAcylation), impacting its localization to the cytoplasm. This modification to YY1 causes cytoplasm retention; therefore, it prevents its transcription factor functionality in the nucleus. The inhibition of O-GlyNAcylation leads to increased apoptosis and indicates the importance of YY1 as a regulator for this cellular process [38,39].

YY1 degradation can also be identified through the regulatory mechanism of Smurf2 E3 ubiquitin ligases. Jeong et al. found that the direct interaction of Smurf2 with YY1 caused poly-ubiquitination and decreased the half-life of YY1, ultimately working as a negative regulator in decreasing YY1 protein levels. The ubiquitin-proteasome-mediated degradation of YY1 further explains the regulatory power of Smurf2 to control the functions of YY1, namely, its transcriptional activity and protein stability [40]. This modification by Smurf, however, can be inhibited by miR-195. The targeted interaction of miR-195 to Smurf2 was found to upregulate YY1 and other proteins, such as vascular endothelial growth factor A (VEGFA) and Snail1, which increased the cell and epithelial mesenchymal transition (EMT) permeability (both of which are characteristics of diabetic retinopathy) [41]. Ultimately, identifying these regulatory factors and pathways is important in that new therapeutic strategies targeting this axis would be beneficial to cancer patients.

## 3. YY1 Expression in Cancer Cells

**Figure 4:**
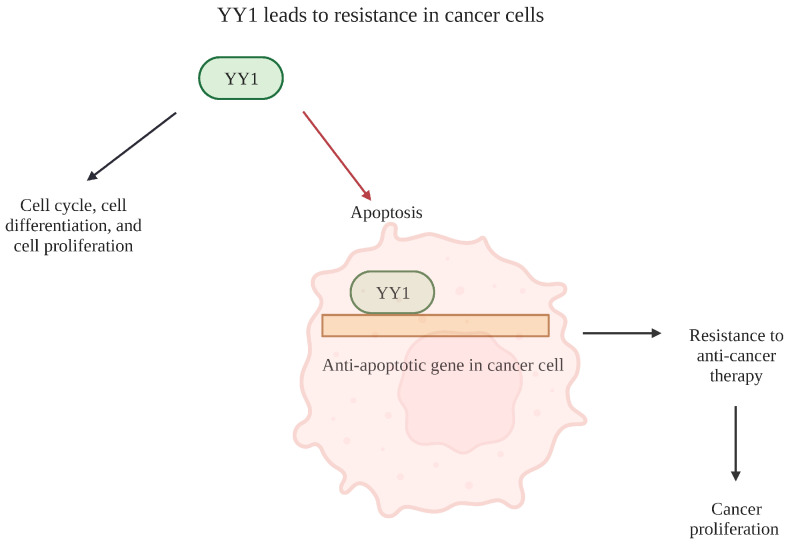
YY1 leads to resistance in cancer cells. Overview detailing the role of YY1 in cellular processes relating to cancer. YY1 is involved with the regulation of several mechanisms regarding cell cycle, cell differentiation and cell proliferation. Specifically, it plays a large role in apoptosis due to its relationship with anti-apoptotic genes in cancer cells. This causes cancer cells to be resistant to apoptosis and, thus, resistant to chemotherapeutic treatments. Ultimately, it can lead to cancer proliferation. Created with BioRender.com (accessed on 1 August 2023).

As a critical regulator of many biological processes, including the cell cycle, differentiation and proliferation, YY1 has profound impacts on DNA repair or apoptosis [42]. The transcription factor YY1 itself is classified as an oncogene and is upregulated in cancer cells. Additionally, YY1 can act as either a positive or negative regulator of other oncogenes involved in the cell cycle depending on its association with other factors [43]. Due to its critical role in tumorigenesis, as well as its involvement with the progression, invasiveness, development and resistance of various cancers, YY1 serves as a prognostic marker and is the focus of cancer therapy as a potential target [44]. YY1′s role as an oncogene is revealed by numerous studies investigating its role in cancer cell lines and is outlined in Figure 4. Below, we outline a few examples.

YY1 plays a dual role in both suppressing and promoting tumor growth [9]. In examining YY1 expression in various types of cancer, many studies have indicated an increased level of YY1 expression in patient samples in contrast to normal samples [45]. Higher YY1 levels are also correlated with enhanced tumor phenotypes; however, this is largely dependent on the tumor context [46]. This is achieved through the activation or repression of genes that are specific to different cancer types, such as the cyclin-dependent kinase inhibitor (*CDKN3*) in pancreatic cancer that YY1 represses or v-erb-b2 avian erythroblastic leukemia viral oncogene homolog 2 (*ERBB2*) in breast cancer that is induced by YY1 [9]. In some cases of cancer, YY1 has been reported to consistently exhibit a point mutation at T372R of YY1′s DNA-binding zinc finger [46]. This mutation leads to the modulation of the insulin secretion pathway through the constant upregulation of the cyclic AMP (cAMP) and Ca^2+^ pathways [47].

YY1-conditional knockout destroys the ability of tumor formations in bladder and breast cancer cell lines through the inhibition of migration and invasion [48,49]. Furthermore, YY1 knockout in colon cancer cells and endometrial cancer cells exhibited apoptosis and suppression of cell proliferation [27,50]. Conversely, ectopic YY1 expression can lead to enhanced tumor invasion activity, migration and tumor activity in gastric cancer, nontumorigenic human mammary MCF-10A cell lines and hepatocyte cells [27,49,51]. This may be perpetuated by the ability of YY1 to promote telomerase activity as seen in laryngeal squamous cell carcinoma (LSCC). In these cell lines, YY1 inhibited growth arrest specific 5 (GAS5) expression, which suppresses p53 stabilization through direct interaction with p300. Ultimately, the decrease in p53 stability leads to further cell proliferation, tumor growth and increased telomere length through telomerase activity, making YY1 an attractive target in oncogenetics [52]. In prostate tumor cells, YY1 as well as its cofactor bromodomain containing proteins (BRD2/4) bind directly to phosphofructokinase and platelet (PFKP) and activates metabolism, thereby, contributing to malignant growth [53].

The expression of YY1 can lead to numerous effects due to its role in the stability and expression of cancer-related genes. Research on breast cancer tumors revealed its role as an oncogenic transcription factor. For example, in breast cancer cell lines, YY1 facilitates p27 ubiquitination and its negative interaction with p27 causes increased tumor growth [49]. In another study, it was found that the YY1 transcription factor activates the activator protein 2-alpha (AP-2α) activity on the oncogenic promoter *ERBB2*, which is found in several breast cancer tumors [54]. YY1 has also been found to have implications with the rate-limiting enzyme PFKP in glycolysis. New research suggests that long non-coding RNAs (lncRNAs) are involved in the regulatory mechanism of YY1 manifesting in a feedback loop. YY1 upregulates the LINC00673 promoter, which targets miR-515-5p and, in turn, increases tumor cell growth by disrupting the breast cancer cell cycle and apoptosis [55].

The role of YY1 and its regulation of various genes has been reported in many reviews and is not the subject of this review. In this paper, we focus on the YY1 regulation of several anti-apoptotic gene products.

## 4. Bcl-2 Family Proteins and IAPs

### 4.1. Bcl-2 Family

The Bcl-2 family of proteins are principal regulators of the process of cell death known as apoptosis. Bcl-2 family proteins are often characterized by the presence of distinct Bcl-2 Homology (BH) domains. The Bcl-2 protein family consists of three subfamilies: pro-apoptotic, anti-apoptotic and BH3-only [56]. The Bcl-2-agonist/killer (BAK) and Bcl-2-associated protein (BAX) are the two primary protein effectors of the pro-apoptotic subfamily [57]. BH3-only proteins also typically have pro-apoptotic functions, and members (such as the BH3 interacting-domain death agonist (BID), phorbol-12-myristate-13-acetate-induced protein1 (NOXA) and p53-upregulated modulator of apoptosis (PUMA)) are able to inhibit anti-apoptotic protein members and activate other pro-apoptotic Bcl-2 proteins [58]. Anti-apoptotic Bcl-2 family members, also known as pro-survival proteins, bind and inhibit pro-apoptotic proteins, such as BAX and BAK. Bcl-2, Bcl-xL and Mcl-1, all of which are notable members of the anti-apoptotic subfamily of Bcl-2 proteins. Due to their known regulatory effects on cell death, anti-apoptotic members Bcl-2, Bcl-xL and Mcl-1 are of importance in cancer therapy as they prevent cell termination in cancer cells and promote the survival of these cancer cells [59]. Particularly, the expression of these anti-apoptotic proteins has been found to be higher in several cancer types; therefore, targeting the Bcl-2 family’s anti-apoptotic proteins may improve cancer therapy or serve to overcome tumor chemotherapy resistance [60].

### 4.2. IAPs

Inhibitors of apoptosis (IAPs) are another group of anti-apoptotic regulators. Distinct from the Bcl-2 family proteins, IAPs are marked by a Baculovirus IAP Repeat (BIR) near the N-terminus [61]. X-linked IAP (xIAP), cellular IAP (cIAP) and survivin are notable IAP members. IAPs exert their pro-survival effect primarily through the interaction with and inhibition of caspases activity. Survivin, along with xIAP, has been shown to inhibit the activation of caspases-3, -7 and -9 [62]. Survivin is often overexpressed in several cancer types and it is also correlated with poor prognosis and worse outcomes [63].

## 5. Overexpression of Anti-Apoptotic Gene Products in Human Cancers

In normal cells, anti-apoptotic and pro-apoptotic factors are highly regulated and the initiation of apoptosis is controlled through a balance of these proteins. The dysregulation of these Bcl-2 family proteins and IAPs is associated with several diseases, including several types of cancer [56,59]. Particularly, the upregulation of anti-apoptotic proteins, such as Bcl-2, Bcl-xL, Mcl-1 and surviving, is associated with cancer progression, tumorigenesis and treatment resistance [64]. Novel strategies to target the upregulation of these anti-apoptotic proteins are a significant opportunity for cancer therapy.

Apoptosis plays a large role in regulating damaged cells during development and can be controlled by various signals within cells. However, the dysregulation of apoptosis may lead to cancer progression, tumor progression and therapeutic tumor resistance. Therefore, it is important to examine the role of these anti-apoptotic factors in relation to cancer and treatment resistance [65]. The previously discussed anti-apoptotic gene products, Bcl-2, Bcl-xL, Mcl-1 and survivin, have been found to be overexpressed in several human cancers. Bcl-2 and Bcl-xL, key regulators of intrinsic apoptosis, are known to prevent apoptosis through the formation of an apoptosome or the prevention of the apoptosis-inducing factor (AIF) presence in the cytoplasm [66,67]. The genetic overexpression of Bcl-2 and Bcl-xL is found in many human cancers. *BCL2* gene amplification is known to occur in follicular lymphoma as a result of chromosomal translocation (14:18) [67,68]. Bcl-xL has also been found to be detected in human cancers with somatic copy-number amplification [69]. Its role in inducing apoptosis and tumor angiogenesis can be traced to the regulation of chemokine interleukin 8 (CXCL8), influencing cell migration [70,71]. Um [72] and Bui et al. [64] outlined Bcl-2 overexpression in prostate cancer, breast cancer, melanomas, small cell lung carcinomas, glioma, neuroblastoma, colorectal cancer and hepatocarcinoma cells. Bcl-xL was found to be overexpressed in breast cancer, prostate cancer, lung cancer and colorectal cancer. Mcl-1, another member of the Bcl-2 family, similarly exhibits cell survival influences on tumor growth through overexpression. Its role in the mitochondrial apoptotic pathway reveals its association with tumorigenesis, drug resistance and poor prognosis [73]. Specifically, Mcl-1 prevents the cytochrome C release from lymphocytes and mitochondrial outer membrane permeabilization (MOMP), which are necessary for cell survival [74]. Therefore, the overexpression of the *MCL1* gene product exhibited by cancer cells results in resistance to anticancer therapies [75]. The overexpression of Mcl-1 is observed in gastric cancer, breast cancer, non-small cell lung cancer (NSCLC), AML and colon cancer cells [64,72].

Finally, survivin, a member of the IAP family, functions as a negative regulator of apoptosis. It does so through the regulation of the nuclear translocation of the AIF, which leads to negative modulation of the caspase-independent apoptosis [76]. In contrast to other IAP members, the survivin homodimer plays a large role in the regulation of mitosis and can inhibit apoptosis by promoting mitosis through the formation of a chromosomal passenger complex (CPC) [77]. This unique role contributes to survivin’s involvement with tumor progression, cancer survival-related signaling pathways and therapeutic resistance [63]. Jaiswal et al. compiled the cancers where survivin was highly expressed: lung, pancreatic, breast, esophageal, ovarian, malignant melanoma, colorectal, hepatocellular, gastric, bladder, AML, acute lymphocytic leukemia and oral cancers [78]. The overexpression of survivin in these cancers contributes to tumorigenesis and poorer prognosis as survivin expression can induce transcriptional changes in the tumor microenvironment (TME) [79].

## 6. YY1 Regulation of Anti-Apoptotic Gene Products (Bcl-2, Bcl-xL, Mcl-1 and Survivin)

**Figure 5:**
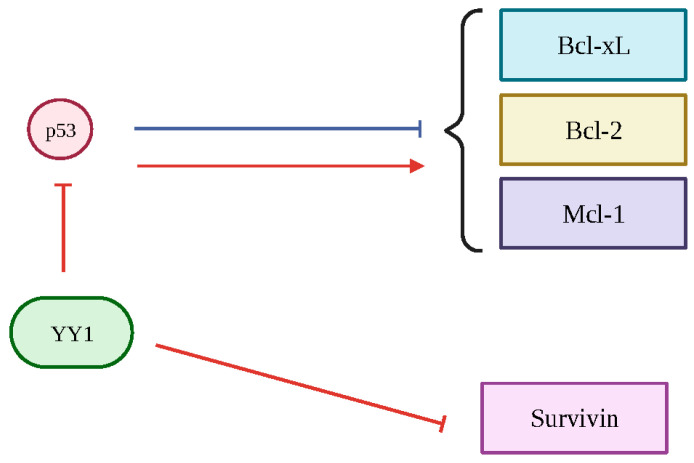
General overview of YY1 regulation of anti-apoptotic genes: Bcl-2, Bcl-xL, Mcl-1 and surviving through its interaction with p53. P53 normally inhibits Bcl-2, Bcl-xL and Mcl-1. YY1 inhibits p53, therefore causing upregulation of Bcl-2, Bcl-xL and Mcl-1. However, YY1 plays a repressive role in relation to survivin. Created with BioRender.com (accessed on 1 August 2023).

Recent perspectives have focused on targeting both YY1 and these anti-apoptotic proteins in order to reverse cancer resistance. Examining the interactions of YY1 and these anti-apoptotic genes may further illustrate their role in cancer pathology and may prove to be beneficial for future targeting strategies. Below, we describe the regulation by YY1 of four anti-apoptotic gene products: Bcl-2, Bcl-xL, Mcl-1 and survivin (Figure 5).

### 6.1. Bcl-2

Martinez-Paniagua et al. (2010) found that the direct inhibition of YY1 is correlated with Bcl-2 and Bcl-xL downregulation [80]. This inhibition is achieved through treatment with Galiximab (anti-CD80 mAb), which inhibits the signaling pathway of NF-κB/YY1/Snail/RKIP/p53 loop, ultimately reversing the resistance to chemotherapy treatments [81]. It was also demonstrated that the inhibition of YY1 through siRNA was also correlated with the downregulation of Bcl-2 and Bcl-xL. Though the exact mechanism of this downregulation was not illustrated, the authors hypothesized two potential contributing pathways. The inhibition of YY1 results in the inhibition of Snail, an inhibitor of RKIP. The inhibition of Snail then leads to the upregulation of RKIP activity, resulting in the inhibition of NF-κB and downstream Bcl-2 and Bcl-xL [82,83]. Additionally, it is likely that the inhibition of YY1 results in the upregulation of p53, thereby downregulating Bcl-2 and Bcl-xL [84].

A subsequent study further confirmed that the NF-κB/YY1/Snail/Bcl-xL loop is associated with drug resistance in B-cell non-Hodgkin lymphoma (B-NHL) cells [85]. The targeting of this regulatory loop via Galiximab resulted in the sensitization to tumor necrosis factor-related apoptosis-inducing ligand (TRAIL)-mediated apoptosis. YY1 is believed to contribute to the regulation of Bcl-xL in this loop directly through putative binding sites on both the Bcl-2 and Bcl-xL promoters [85].

Another study by Vivareli et al. further investigated the implications of YY1 on similar Bcl-2 family members in colorectal cancer (CRC) cells. The findings revealed that increased resistance to 5-Fluorouracil-induced cytotoxicity in CRC cells was due to the silencing of YY1. Furthermore, YY1 suppression and *BCL2L15* downregulation were found in these cells, marking YY1 and *BCL2L15* prognostic markers for CRC patients [86]. Additionally, it was found that phosphorylation at serine-118 (S118), located in the transactivation domain of YY1, may be involved in controlling the expression of the anti-apoptotic genes of the Bcl-2 family or caspase inhibitors. S118 is a site for casein kinase II subunit alpha (CK2α) phosphorylation and protects against caspase 7 cleavage despite apoptotic signals from Bcl-2, contributing to tumorigenesis [87].

In a recent study, YY1 promoted cancer progression in human melanomas [88]. Zhou and colleagues showed that the overexpression of YY1 increases cell survival in melanoma cells, and the knockout of YY1 was associated with decreases in cell proliferation and migration. YY1 was determined to have this anti-apoptotic effect primarily due to the regulation of p53 and downstream Bcl-2. Several studies have demonstrated the negative regulatory relationship between YY1 and p53 [84,89,90]. Further, p53 has been demonstrated to inhibit several anti-apoptotic proteins, including Bcl-2, Bcl-xL and Mcl-1 [52,91]. Zhou and colleagues suggested that the knockdown of YY1 promotes apoptosis through the YY1/MDM2/p53/Bcl-2 pathway, as p53 is upregulated and downstream anti-apoptotic Bcl-2 members are downregulated [88].

### 6.2. Bcl-xL

**Figure 6:**
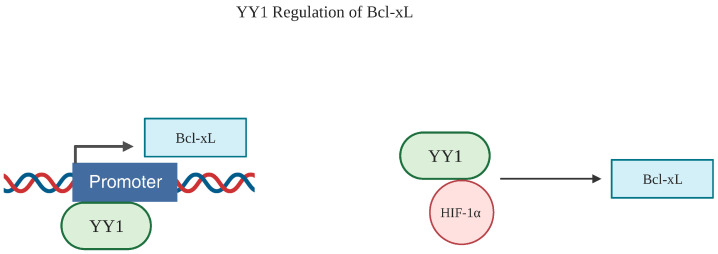
YY1 regulation of Bcl-xL. YY1 regulates Bcl-xL through via different mechanisms. Through a direct transcriptional relationship, YY1 binds to a potential promoter region in the −425 position of the Bcl-xL gene. Another mechanism involves the stabilization of HIF-1α through binding with YY1, which ultimately upregulates Bcl-xL. Created with BioRender.com (accessed on 1 August 2023).

Several groups have reported the YY1 regulation of Bcl-xL via multiple means. One study indicated a direct transcriptional relationship between YY1 and Bcl-xL. Morales-Martínez and Vega located a potential YY1 binding site at the -425 position in the Bcl-xL promoter region [92]. Similarly, the hypoxia-inducible factor-1α (HIF-1α) was also found to be able to regulate Bcl-xL and is also itself regulated by YY1. One study showed that HIF-1α is stabilized by YY1, and the silencing of YY1 reduced cancer progression under hypoxic conditions [93]. These investigators determined that the stabilization of HIF-1α by YY1 was related to p53 signaling. Furthermore, several studies have indicated that increased HIF-1α is linked with the upregulation of Bcl-xL, and HIF-1α has also been shown to regulate Bcl-xL at the level of transcription [94,95,96] (Figure 6).

**Figure 7:**
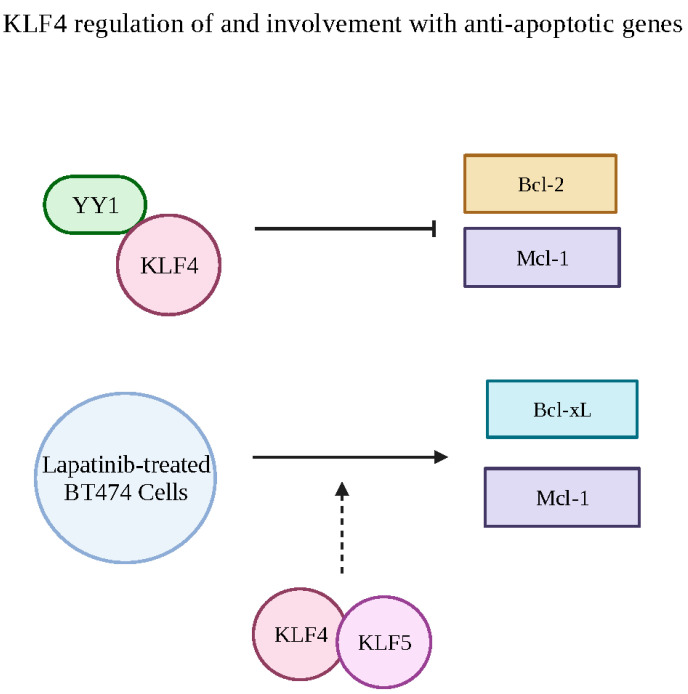
KLF4 interaction and regulation of anti-apoptotic genes. There are two YY1-binding sites on the promoter region of KLF4, which may have implications with the expression of anti-apoptotic genes. Increased KLF4 expression has been known to reduce Bcl-2 and Mcl-1 expression. Therefore, it is proposed that YY1 acts as an activator, upregulating KFL4, which would downregulate Mcl-1 and Bcl-1. Additionally, in lapatinib-treated BT474 cells, Bcl-xL and Mcl-1 were found to be upregulated in a KLF4/5-dependent manner. Created with BioRender.com (accessed on 1 August 2023).

In another study, YY1 was reported to transcriptionally regulate Kruppel-like Factor 4 (KLF4), another molecule that has been shown to regulate Bcl-xL [97]. Morales-Martinez and colleagues found two putative YY1-binding sites in the KLF4 promoter [97]. Furthermore, several studies have identified several regulatory connections between KLF4 and multiple anti-apoptotic proteins. Early studies showed that the increased expression of KLF4 resulted in decreased Bcl-2 levels [98]. In the same study, Li and colleagues found a putative KLF4-binding element at the nucleotide position −177/−163 in the promoter region of Bcl-2 [98]. Another study showed that the combination of increased KLF4 expression with apigenin reduced Bcl-2 and Mcl-1 expressions while increasing the expression of several pro-apoptotic Bcl-2 members [99]. Interestingly, a further study showed Mcl-1 and Bcl-xL protein and mRNA levels were reduced in KLF4/KLF5-deficient breast cancer cells [100]. In the same study, Mcl-1 was upregulated during lapatinib treatment through KLF4/KLF5 induction, contributing to treatment resistance. Similarly, Bcl-xL and Mcl-1 were upregulated in a KLF4-dependent manner in lapatinib-treated BT474 cells [100] (Figure 7).

YY1 has been shown to regulate Bcl-xL through the regulation of the miRNA let-7a, an inhibitor of Bcl-xL [34,101]. It was demonstrated that YY1 is activated downstream of the SDF-1α/CXCR4 axis, and that YY1 functions as a negative regulator of let-7a. In AML cells, Chen and colleagues suggested that this CXCR4/YY1 pathway contributed to resistance through the downregulation of let-7a, leading to the activation of Bcl-xL [34].

Bcl-xL also plays a role in chemoresistance, and modulating its levels is known to promote cancer cell survival in the presence of chemotherapy drugs [102]. YY1 may play an indirect synergistic role in this resistance with NF-κB, which is known to control Bcl-xL transcription [103]. In the study by Vega et al., treatment with Rituximab (anti-CD20 mAb) of B-NHL cell lines was found to inhibit NF-κB activity, downregulating Bcl-xL and sensitizing the cells to drug-induced apoptosis. NF-κB also controls YY1 transcription activity, and Rituximab treatment resulted in the inhibition of YY1 activity [104]. This increased sensitization to drug-induced apoptosis, suggesting that chemoresistance is caused by different mechanisms involving YY1 and Bcl-xL.

### 6.3. Mcl-1

Mcl-1 is among the anti-apoptotic family found to be a regulator of apoptosis in many malignant cells [105]. While Bcl-2 family members are often involved in translocations, Mcl-1 mutations in cancer are rare, yet it is one of the highest amplified genes found in human cancer cells [69]. A recent study by Shao et al. investigated the role of YY1 and its influence on colorectal cancer. They found that anti-apoptotic Mcl-1 may play a role in tumor cell progression through a p53-dependent manner as YY1 acts to upregulate Mcl-1 and downregulate pro-apoptotic caspases -3,-7 and -9 [106]. Ultimately, this interaction indicates YY1′s ability to suppress apoptosis through its regulation of anti-apoptotic Mcl-1.

Another study by Martinez-Panaigua et al. demonstrated the effects of Mcl-1 and YY1 inhibition on Obatoclax-induced sensitization to TRAIL apoptosis [107]. In their findings, they revealed that the death receptor 5 (DR5) modulates the expression and activity of Mcl-1 and YY1 through the inhibition of NF-κB. Therefore, altering the apoptotic pathways may allow for TRAIL resistance to be overcome and to promote cell death in cancer cells [107]. They determined that YY1 is likely to regulate Mcl-1 through an NF-κB/YY1 regulatory loop and in which the inhibition of YY1 was shown to contribute to the reversal of TRAIL apoptosis, in part, through the downregulation of NFκB and downstream inhibition of Mcl-1. In the same study, YY1 was suggested to also potentially downregulate Mcl-1 through a more direct means, though the exact mechanism of this downregulation was not determined [107]. Additionally, YY1 and Mcl-1 may also contribute to the regulatory effects of several miRNAs [108]. miR-29 has been demonstrated to inhibit both YY1 and Mcl-1. These findings suggested that the Mcl-1 reduction downstream of YY1 may contribute to the observed inhibition of Mcl-1 by miR-29 [107,108]. Additionally, a similar effect is shown by miR-181. miR-181 expression is associated with the repression of Bcl-2 and Mcl-1. Morales-Martinez and Vega suggested that YY1 may contribute to the regulatory effects of miR-181 on Bcl-2 and Mcl-1 [108].

### 6.4. Survivin

Survivn, encoded by the baculoviral IAP repeat-containing 5 (*BIRC5*) gene, is an essential protein for the regulation of cell cycle progression, cell division and the inhibition of apoptosis [109]. Survivin is highly expressed in the G2-M phase cell cycle. Additionally, survivin is often upregulated in cancer cells, making it an attractive potential therapeutic target [110]. Survivin has been found to be transcriptionally regulated by YY1. Specifically, YY1 was found to strongly bind to the *BIRC5* promoter, which was confirmed through both JASPAR predictive and CHIP-seq data [109]. The study by Galloway et al. confirmed YY1′s involvement in survivin repression, and the knockdown of YY1 released the BIRC5 promoter and may lead to an increase in promoter activity above normal levels. YY1 acts as a transcription factor through the direct binding of the survivin core promoter, leading to transcriptional repression [111]. Two putative YY1-binding sites have been identified in the *BIRC5* promoter. Both binding sites were located in the first 230 bp region, with the strongest protein–protein interactions being established at the most proximal side of the BIRC5 promoter. In a follow-up study by Galloway et al., it was found that YY1-binding sites overlap with serine protease 1 (Sp1), a necessary transcription factor for the expression of basal survivin, and can act as either an activator or repressor of survivin transcription depending on the circumstance [112]. They also found that the localization of survivin to the extracellular space may be modulated by either YY1 overexpression or knockdown, though further studies are needed to investigate this mechanism as a therapeutic approach for tumors [112].

Survivin is an important factor in cancer due to its role in preventing the apoptosis of tumor cells. Vivarelli et al. demonstrated the influence of overexpressing survivin in Non-Hodgkin’s lymphoma (NHL) cancer patients, showing that high survivin levels were correlated with poor prognosis [109]. Further, in the same study they found that YY1′s overexpression in hematological tumors influenced its downstream target, survivin. Through examining YY1 knock-down in Raji cells, survivin levels were significantly downregulated, indicating a positive regulation between YY1 and survivin expression. Therefore, both YY1 and survivin serve a pro-tumorigenic role in aggressive B-NHLs, particularly in B-cell lymphomas (BLs), and survivin is likely a good potential diagnostic and prognostic biomarker for chemotherapy responses in patients with NHL [109]. While this study demonstrated YY1′s inhibitory regulation of survivin transcription, there is evidence that YY1 may act as a positive regulator depending on the interaction of its cofactors in different circumstances. Affar et al. found that, in mice YY1 knockdown models, survivin levels were significantly decreased. The depletion of YY1 led to the downregulation of the RNAi-mediated inhibition of survivin, along with the modulation of p53 checkpoint resulting in cell cycle arrest in G1 [113]. Zhang et al. similarly found that YY1 expression was positively correlated with the expression of survivin [114]. Through YY1′s activation of the Wnt signaling pathway, the upregulation of the protein fibroblast growth factor 4 (FGF4) and survivin increased in CRC cells, leading to a stimulatory role in tumorigenesis [114]. YY1′s dual role as a transcription factor can be explained by the cellular contexts or mutated binding sites within the *BIRC5* promoter of cancer cells, impacting the binding of YY1 [43].

A summary of the different pathways through which YY1 regulates each apoptotic factor discussed is outlined in Table 1.

## 7. YY1-Mediated Resistance to CD8 Cytotoxic T Cells and Chemotherapeutic Drugs

**Figure 8:**
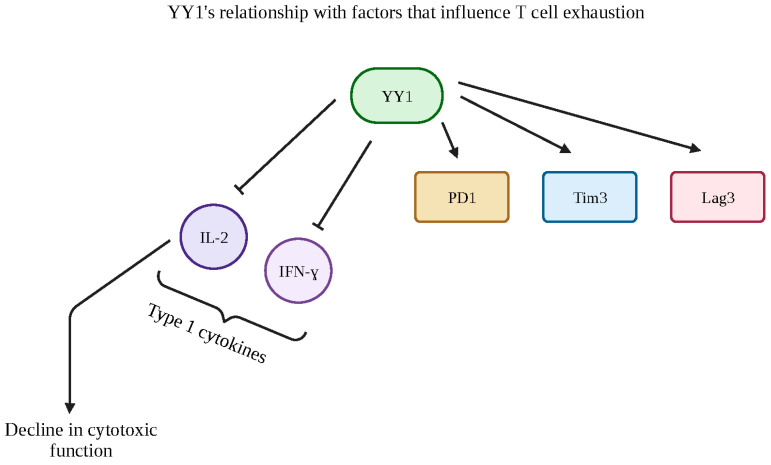
YY1′s relationship with factors that influence T-cell exhaustion. Schematic diagram that depicts YY1′s implications with cytotoxicity in order to understand how to reverse chemotherapeutic resistance. YY1 is closely linked with T-cell exhaustion through its positive regulation of checkpoint inhibitors PD1, Tim3 and Lag 3 and its negative regulation of the type 1 cytokines IL-2 and IFN-ɣ. The downregulation of these cytokines is primarily related to a decline in cytotoxic functions, revealing elevated YY1 levels to be a biomarker in tumor-infiltrating lymphocytes. Created with BioRender.com (accessed on 1 August 2023).

Cancer immune surveillance failure can be attributed to the process of T cell exhaustion. T cells normally assist in eradicating malignancy or infections; however, dysfunctional T cells, which lead to exhaustion, may cause resistance and the survival of tumor cells [115,116]. Cytokine deprivation and checkpoint receptor action are critically important in the T-cell exhaustion model, and Balkhi et al. found that YY1 has the ability to regulate these elements [115]. This study identified a YY1-centered mechanism that is correlated with T-cell exhaustion: YY1 positively regulates the programmed cell death protein 1 (PD-1), T-cell immunoglobulin mucin-3 (Tim3), and lymphocyte activation gene (Lag3) checkpoint inhibitors but negatively regulates IL-2 and interferon gamma (IFN-ɣ), both of which are type I cytokines. This IL-2 downregulation is a large factor in the decline in cytotoxic function, as elevated YY1 levels were found in tumor-infiltrating lymphocytes. Thus, the model of a two-signal stimulation feedback loop was developed: VCD3/CD28—p38MAPK/JNK—YY1—exhaustion [115] (Figure 8).

Drug-induced apoptosis, accomplished by the nitric oxide (NO) sensitization of tumor cells, resulted from inhibition of YY1 and its anti-apoptotic gene products Bcl-2 and Bcl-xL [117,118]. Montecillo-Aguado et al. identified that KLF4, overexpressed in NHL, was under the transcriptional control of YY1 and is a resistant factor in drug-induced apoptosis [119]. They identified the resistant axis in NHL: NF-κB/YY1/KLF4/Bcl-xL/Mcl-1 [119]. Its anti-apoptotic gene products (Bcl-xL and Mcl-1) serve as prognostic biomarkers and promising therapeutic targets as their overexpression influences drug resistance [100]. A recent study by Holthof et al. investigated the implications of chimeric antigen receptor (CAR) T-cell-based therapy for multiple myeloma (MM) and the cellular resistance to this therapy [120]. They found that the upregulation of survivin and Mcl-1 in MM cells protected MM cells against cytotoxic T cells (CTL) and natural killer (NK) cells [120]. Furthermore, Martinez-Panaigua et al. (2010) identified that treatment with Galiximab modulating the NF-κB/YY1/Snail/RKIP/p53 loop, inhibited each factor in this pathway and ultimately exhibited downstream inhibition of Bcl-2 and Bcl-xL, reversing resistance [80]. Therefore, identifying the inhibitors of YY1 is critical for future therapeutic targets that aim to induce the sensitization of tumor cells to cell death and decrease resistance.

## 8. Reversal of Resistance by Targeting YY1

Due to the role of YY1 as a key regulator in cellular processes, such as survival, proliferation, invasion and metastasis, it plays a large role in the drug-resistance of cancer cell lines [121]. Therefore, the inhibition of YY1 through indirect and direct targets could lead to the reversal of resistance [3]. YY1 inhibition can be achieved through several different factors, which are briefly discussed. Firstly, siRNA YY1 acts as a YY1 inhibitor through the upregulation of Let7a DNA fragments to which YY1 binds. Let7a targets Bcl-xL and acts as a tumor suppressor, so the siRNA YY1 blocks YY1 from suppressing Let7a and allows for chemosensitivity in tumor cells [121]. MicroRNAs also play a role in the inhibition of YY1 as Zhang et al. reported that miR-7 binds to *YY1* 3′UTR, ultimately leading to the downregulation of YY1 in CRC, inducing apoptosis [114]. Additionally, miR-181 was also found to inhibit cell proliferation and negatively regulate YY1 [122]. Therefore, utilizing miRNA to inhibit YY1 may lead to decreased tumorigenesis and could overcome resistance. NO may also help to resolve resistance through its regulation of anti-apoptotic gene products [64]. It has been found that, for prostate carcinoma cell lines that are drug-resistant, treatment with NO-donor DETANONOate reversed the resistance in combination with CDDP, a chemotherapeutic drug. This treatment caused tumor cells to be sensitized to CDDP-induced apoptosis in a concentration-dependent manner [118]. Finally, the indirect pathway of NF-κB/YY1/Snail/RKIP circuitry can be targeted to inhibit YY1. This is achieved through inducing RKIP, which would cause the inhibition of NF-κB, Snail and YY1, helping to increase the sensitization of cells resistant to drug-induced apoptosis [24]. The disruption of the NF-κB/YY1/Snail/RKIP loop in cancer cells reverses chemoresistance due to the inverse relationship between YY1 and RKIP.

The introduction of guide RNAs (gRNAs), designed to directly target the *YY1* gene in cancer cells, is a promising new therapy. The CRISPR/Cas9-mediated knockout acts on cancer cells, and it specifically targets and interferes with the *YY1* gene. This is achieved through the Cas9 nuclease acting to induce DNA cleavage as well as gene disruption. Overall, this genetic knockout therapy may provide a long-term solution due to its long-lasting effects on the inhibition of YY1. Other signaling pathways that activate YY1, such as MAPK/ERK or P13K/AKT, can be targeted for inhibition, leading to YY1 downregulation and a reduced expression of the gene. Furthermore, transcription factors, such as NF-κB and E2F, which act as positive regulators of YY1, may be targeted for the indirect inhibition of YY1 expression.

**Figure 9:**
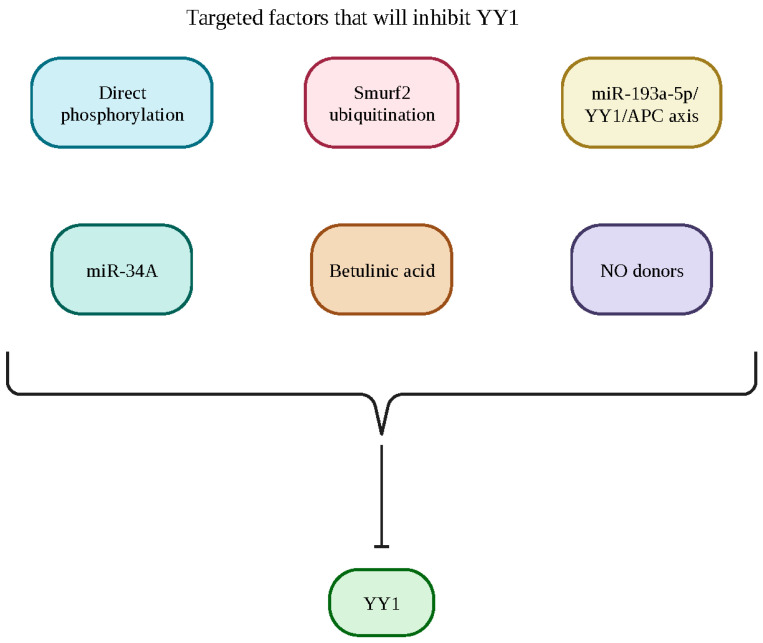
Summary of the different potential targets that can inhibit YY1 and its downstream effects. These targets include direct phosphorylation, ubiquitination by Smurf2, the axis miR-193a-5p/YY1/APC, miR-34A expression, betulinic acid and NO donors. Created with BioRender.com (accessed on 1 August 2023).

**Figure 10:**
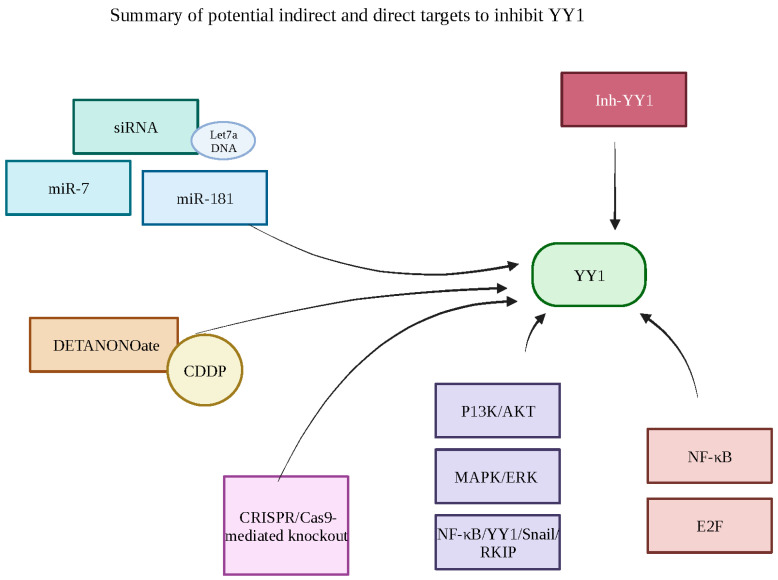
Summary of potential indirect and direct targets to YY1. Overview of the potential factors that may be targeted in order to inhibit YY1 and reverse cancer cell resistance to drug-induced apoptosis. The siRNA and Let-7aDNA, miR-7 and miR-181 are all potential targets that, when inhibited, downregulate YY1. The NO donor DETANONOate, along with the chemotherapeutic drug CDDP, can be targeted as well. The pathways involved in regulating YY1 include MAPK/ERK, P13K/AKT and NF-κB/YY1/Snail/RKIP and the transcription factors NF-κB and E2F may be the subject of the indirect inhibition of YY1. Finally, using gRNA, the CRSPR/Cas-9-mediated knockout or the novel synthetic Inh-YY1 can be utilized to directly and specifically target YY1. Created with BioRender.com (accessed on 1 August 2023).

The recent development of a small synthetic inhibitor of YY1 (Inh-YY1), structurally known as [(5-nitro-2-methyl-1-3-dioxan-5-yl) methanol], is currently in preparation to bridge the gap and need for a specific inhibitor of YY1. Ideally, the inhibition of YY1 will lead to the sensitization of resistant tumor cells to drug-induced apoptosis and encourage tumor growth inhibition. The specific YY1 inhibitor (Inh-YY1) inhibited YY1-DNA and can be traced to the activator activity on the *MDR1* gene and the repressor activity on the *Fas* gene in a study conducted by Lopez-Perez et al. (unpublished). In this study, they investigated three leukemic cell lines expressing varying amounts of YY1 [1] marrow-derived [2] T lymphoblast and [3] B lymphoblast by exposing them to Inh-YY1 and chemotherapeutic drugs. They found that all cell lines showed sensitization to apoptosis, with the concentration of Inh-YY1 and chemotherapeutic drugs differing depending on the YY1 levels in each cell lineage. The inhibitory mechanism behind Inh-YY1 is achieved through the diastereomer of Inh-YY1 interaction at the DNA-binding domain of YY1. This newly synthesized inhibitor was specific in targeting tumor cells with YY1; however, further developments and studies are necessary to ensure the inhibitor’s effectiveness in in vitro and murine models (Lopez-Perez et al.; unpublished). Additionally, YY1 siRNA and the chemical inhibitor Kenpaullone result in tumor cell sensitization to drug-induced apoptosis [119]. Bonavida has reviewed targeted factors that will specifically inhibit YY1 [121]. The identified the factors that induce YY1 inhibition, including direct phosphorylation, ubiquitination by Smurf2, the axis miR-193a-5p/YY1/APC, miR-34A expression, betulinic acid and NO donors, and miR-181 [122] (Figure 9 and Figure 10).

## 9. Reversal of Resistance by Targeting Anti-Apoptotic Gene Products

Due to the fact that anti-apoptotic proteins are highly associated with poor prognosis and cancer resistance, the direct therapeutic targeting of these members has become an important new strategy. Targeting Bcl-2 family members has been thoroughly reviewed [123,124,125], though we discuss it briefly in this paper. Currently, BH3 mimetics are the primary strategy for Bcl-2 family inhibition. Several BH3 mimetics exist, including ABT-737, ABT-263, Obatoclax and ABT-199 (Venetoclax). Venetoclax remains one of the forefront BH3 mimetics. Venetoclax has seen considerable success clinically and is an approved treatment for several hematological cancers [123]. Another novel Bcl-2 family targeting strategy is the use of proteolysis-targeting chimeras (PROTACs). PROTACs selectively degrade protein targets and have advantages over standard treatments due to their ability to directly and specifically degrade their targets. Early studies have shown success for Bcl-xL PROTAC [126,127,128], and similar Mcl-1 and Bcl-2 PROTACs are also in the early stages of study [129]. Further, there are also several strategies for targeting survivin. Primary strategies for survivin targeting focus on inhibiting its interactions with other proteins, though other strategies involve decreasing survivin gene transcription [130,131]. LQZ-7 and LQZ-7F are one of the first inhibitors of survivin dimerization [132]. There are also several novel strategies for survivin targeting utilizing nanotechnology as reviewed in George et al. [130].

The targeting of these anti-apoptotic proteins is a novel strategy for reversing resistance in cancers with the overexpression of these proteins. Due to the fact that resistance to apoptosis is highly connected to resistance of many conventional therapies, targeting the proteins that promote survival is very likely to be effective. Additionally, the use of these targeting strategies in addition to other novel treatments may prove to be synergistic. It would be ideal to target treatment resistance at several levels and novel drug combinations should be studied.

## 10. Discussion

Our findings highlight that the transcription factor YY1 has the potential to promote or repress oncogenic proliferation in cancer cells depending on several factors. The regulation of YY1 expression can be traced to the transcription level through autoregulation, the NF-κB/YY1/microRNA-10a circuit and the NF-κB/Snail/YY1/RKIP regulatory loop [24,109]. Post-transcriptionally, YY1 is regulated by direct acetylation, phosphorylation or methylation [25,26,27]. Further signaling pathways involving microRNAs have been found to directly target YY1 expression, and the stability of YY1 was found to be implicated with Smurf2 [30,33].

In cancer cells, YY1 has the ability to promote and repress tumor growth [43]. Several studies have found that higher levels of YY1 are associated with tumor growth, while other studies found that lower levels of YY1 may help to combat tumor invasion activity [9]. Importantly, YY1 plays a significant role in the regulation of anti-apoptotic gene products: Bcl-2, Bcl-xl, Mcl-1 and survivin [80,112]. These anti-apoptotic genes are of interest as their overexpression is associated with cancer therapeutic resistance as well as promoting cancer proliferation and cancer cell survival [66,67]. The inhibition of YY1 is correlated with Bcl-2 and Bcl-xL inhibition and YY1 inhibition upregulates p53, resulting in Bcl-2 suppression through the p53 pathway, thereby promoting apoptosis [84]. YY1 inhibition was also found to downregulate Bcl-2 and Bcl-xL through the NF-κB/YY1/Snail/RKIP signaling loop [82,83]. Additionally, Bcl-xL inhibition by YY1 through the modulation of NF-κB activity was also found to help to sensitize cells to treatment [102,103,104]. Further, YY1 was found to have binding sites on both Bcl-2 and Bcl-xL promoter regions, and likely transcriptionally upregulates both anti-apoptotic genes [92]. Other factors, such as KLF4 and miRNA let-7a, are also able to alter Bcl-xL expression when modulated by YY1 (34,99–101). Furthermore, Mcl-1 is regulated by YY1 in a p53-dependent manner, through DR5, miR-29 and miR-181 [107,108]. Finally, survivin is transcriptionally regulated by YY1 and can act as either an inhibitor or promoter due to different interaction factors [109,113]. Studies have found that suppressing YY1 downregulates survivin significantly, while other studies found that YY1 knockdown releases survivin promoter activities through strong binding interactions with its promoter region [109,110,112]. These outcomes may be explained by the TME or the specific cell context and require further investigation.

YY1 also has implications with T-cell exhaustion, attributing its role in resistance to cytotoxic T cells [116]. YY1 regulates several checkpoint inhibitors: PD1, Tim3 and Lag3, but negatively regulates type I cytokines IL-2 and IFN-ɣ [115]. With a decline in cytokines, the cytotoxic function is decreased and can increase tumor cell resistance. Therefore, the inhibition of YY1 will allow for its downstream anti-apoptotic gene products to be downregulated and allow for drug-induced apoptosis. Some known inhibitors, such as siRNA, miR-181 and the NO-donor DETANONOate, and the NF-κB/YY1/Snail/RKIP circuitry are able to indirectly block YY1 activity [121]. Additionally, gRNAs for the direct target of YY1 are specialized in the interference of the gene. The CRISPR/Cas-9-mediated knockout or the novel Inh-YY1 are both promising in their abilities to achieve sensitivity to cancer drug therapy.

## 11. Conclusions and Future Perspectives

The present paper reviewed novel perspectives on the relationship between the overexpression of YY1 in cancer and the expression of anti-apoptotic proteins, offering valuable insights into the role of YY1 in regulating resistance to apoptosis-induced cytotoxic therapies. Various mechanisms were discussed by which YY1 regulates the expression of the anti-apoptotic proteins, Bcl-2, Bcl-xl, Mcl-1 and survivin, both directly at the transcriptional and post-transcriptional levels and indirectly. We also delved into the functional consequences of the YY1-mediated regulation of these anti-apoptotic proteins and highlighted the role of YY1 in conferring resistance to apoptosis and, hence, promoting tumor cell survival and metastasis and significantly contributing to cancer resistance in various cancer types to both chemotherapy and immunotherapy.

This report also discussed the potential clinical significance of YY1 and anti-apoptotic proteins as potential targets in cancer treatment. It also explored various therapeutic strategies that have been reported to modulate YY1 expression or activity, including RNA-based approaches, small molecules, peptides and a small-molecule-specific YY1 inhibitor. These various YY1 inhibitors are discussed as significant chemo-immuno-sensitizers when used in combination to treat resistant cancer cells. It was also discussed the preference of targeting YY1 as its inhibition will be manifested by various consequential effects, not only in the inhibition of anti-apoptotic proteins, but also in the inhibition of cell proliferation, invasion and cancer stem cells.

In this report, we only focused on very few anti-apoptotic proteins and further research must address other anti-apoptotic proteins as well as the regulation of pro-apoptotic proteins. We addressed the challenges we are facing with the clinical application of targeting YY1 and more research is needed to validate its potential clinical application. Clearly, it is also important to identify biomarkers and the patient subgroups who will benefit from targeting YY1 or anti-apoptotic proteins.

In conclusion, this report provided a comprehensive analysis of the current understanding of the interplay between YY1 and anti-apoptotic proteins in cancer. It highlighted the significance of YY1 in promoting cancer cell survival and therapy resistance via the YY1 regulation of anti-apoptotic proteins. It underscored the potential of YY1 and anti-apoptotic proteins as therapeutic targets in cancer treatment and urgently suggested further research to translate the findings into clinical applications in cancer patients.

## Figures and Tables

**Table 1:** YY1 regulation of anti-apoptotic factors through various pathways.

Anti-Apoptotic Factor	Regulatory Pathway Involved	Type of Regulation	References
Bcl-2	YY1/Snail/RKIP/NF- κB loop	Positive	[81,82,83]
	YY1/MDM2/p53	Positive	[84,88,89,90]
	YY1/KLF4	Negative	[97,98]
	miR-181/YY1	Negative	[108]
Bcl-xL	NF-κB/YY1/Snail	Positive	[85]
	YY1/HIF-a	Positive	[94,95,96]
	YY1/KLF4/KLF5	Positive and Negative	[97,99,100]
	CXCR4/YY1/let7a	Positive	[34]
Mcl-1	YY1/p53	Positive	[106]
	NF-κB/YY1 loop	Positive	[107]
	miR-29/YY1	Negative	[107]
	miR-181/YY1	Negative	[108]
Survivin	Wnt/FGF4	Positive	[114]
	p53	Positive	[113]
	RNAi	Positive	[113]

## Data Availability

No new data were created or analyzed in this study. Data sharing is not applicable to this article.

## Abbreviations

AML, acute myeloid leukemia; Bcl-2, B-cell lymphoma 2 protein; Bcl-xL, B-cell lymphoma extra-large; BH, Bcl-2 homology; B-NHL, B-cell non-Hodgkin lymphoma; CRC, colorectal cancer; CXCR, Cxc motif chemokine receptor; HDAC, histone deacetylase; HIF-1α, hypoxia inducible factor-1α; IAPs, inhibitors of apoptosis; IL, interleukin; KD, knockdown; KRAS, Ki-ras2 Kristen rat sarcoma viral oncogene homolog; KLF4, Kruppel-like factor 4; Mcl-1, myeloid cell leukemia 1; MDR1, multidrug resistance 1 gene; miR, microRNA; MM, multiple myeloma; NF-κB, nuclear factor-kappa B; PROTACs, proteolysis-targeting chimeras; RAF, rapidly accelerated fibrosarcoma; RKIP, Raf kinase inhibitory protein; siRNA, small interfering RNA; TF, transcription factor; TRAIL, tumor necrosis factor-related apoptosis-inducing ligand; YY1, Ying Yang 1.
